# Early Treatment with Monoclonal Antibodies or Convalescent Plasma Reduces Mortality in Non-Vaccinated COVID-19 High-Risk Patients

**DOI:** 10.3390/v15010119

**Published:** 2022-12-30

**Authors:** Laura Thümmler, Monika Lindemann, Peter A. Horn, Veronika Lenz, Margarethe Konik, Anja Gäckler, Kristina Boss, Fotis Theodoropoulos, Vasiliki Besa, Christian Taube, Thorsten Brenner, Oliver Witzke, Adalbert Krawczyk, Hana Rohn

**Affiliations:** 1 Department of Infectious Diseases, West German Centre of Infectious Diseases, University Medicine Essen, University Hospital Essen, University Duisburg-Essen, 45147 Essen, Germany; 2 Institute for Transfusion Medicine, University Medicine Essen, University Hospital Essen, University Duisburg-Essen, 45147 Essen, Germany; 3 Department of Nephrology, University Hospital Essen, University Duisburg-Essen, 45147 Essen, Germany; 4 Department of Pneumology, University Medicine Essen—Ruhrlandklinik, University Duisburg-Essen, 45147 Essen, Germany; 5 Department of Anesthesiology and Intensive Care Medicine, University Hospital Essen, University Duisburg-Essen, 45147 Essen, Germany; 6 Institute for Virology, University Hospital Essen, University Duisburg-Essen, 45147 Essen, Germany

**Keywords:** SARS-CoV-2, convalescent plasma, monoclonal antibody treatment, Coronavirus Disease 19

## Abstract

Vulnerable patients such as immunosuppressed or elderly patients are at high risk for a severe course of COVID-19 upon SARS-CoV-2 infection. Immunotherapy with SARS-CoV-2 specific monoclonal antibodies (mAb) or convalescent plasma represents a considerable treatment option to protect these patients from a severe or lethal course of infection. However, monoclonal antibodies are not always available or less effective against emerging SARS-CoV-2 variants. Convalescent plasma is more commonly available and may represent a good treatment alternative in low-income countries. We retrospectively evaluated outcomes in individuals treated with mAbs or convalescent plasma and compared the 30-day overall survival with a patient cohort that received supportive care due to a lack of SARS-CoV-2 specific therapies between March 2020 and April 2021. Our data demonstrate that mAb treatment is highly effective in preventing severe courses of SARS-CoV-2 infection. All patients treated with mAb survived. Treatment with convalescent plasma improved overall survival to 82% compared with 61% in patients without SARS-CoV-2 targeted therapy. Our data indicate that early convalescent plasma treatment may be an option to improve the overall survival of high-risk COVID-19 patients. This is especially true when other antiviral drugs are not available or their efficacy is significantly reduced, which may be the case with emerging SARS-CoV-2 variants.

## 1. Introduction

Since its initial description in late 2019, the severe acute respiratory syndrome coronavirus 2 (SARS-CoV-2) spread over the world and caused a pandemic causing enormous health and socioeconomic implications. The symptoms of a SARS-CoV-2 infection range from cold-like symptoms accompanied by cough and fever to severe pneumonia or disseminated infection that may be fatal [1]. Patients with pre-existing chronic conditions such as cardiovascular disease, obesity, diabetes, cancer, chronic kidney or lung disease, and compromised immune status are at particularly high risk for COVID-19-related morbidity and mortality [2,3,4,5]. During the early pandemic, COVID-19 patients received supportive medical care because antiviral treatment was not yet available. Convalescent plasma from recovered patients contains neutralizing antibodies and thus may represent a beneficial treatment option for viral infections. Immunotherapy of potentially lethal infections, lacking specific preventive and therapeutic options, with passive immunization with convalescent plasma was successfully used in other viral diseases such as SARS, Ebola or influenza A (H5N1) [6,7,8,9]. The experimental treatment of SARS-CoV-2 infection with convalescent plasma has therefore been proposed particularly in the vulnerable cohort of high-risk patients [10,11,12,13,14,15]. Initial studies reported an improvement in the clinical condition and survival in patients undergoing plasma treatment [16,17,18,19]. However, recent studies with a large number of patients with varying underlying health conditions have failed to demonstrate the significant clinical benefit of convalescent plasma in COVID-19 patients [20,21]. Notably, two large randomized, double-blind, placebo-controlled trials studies examined the efficacy of early-administered convalescent plasma in elderly patients and reported a reduced progression of COVID-19 [22,23]. Within the progress of the pandemic, major advances have been achieved in understanding, prevention, and treatment of the disease. COVID-19 can be subdivided in three different phases: An early phase with viremia, fever and cough (phase I), a pulmonary vascular disease (phase II) and a hyperinflammatory syndrome (phase III) [13]. Notably, the viral replication plays an important role in the early stage of infection. Obviously, the onset of passive antibody treatment must occur in a timely manner after infection to be effective [24]. Consequently, antiviral interventions are most effective during the early phase of the disease, whereas immunoregulatory treatment occurs effective during the hyperinflammatory stage [13]. The direct antiviral drugs remdesivir, molnupinavir and nirmatrelvir/ritonavir have been shown to be effective for preventing severe forms of COVID-19 if administered early [25]. The approval of neutralizing monoclonal antibodies (mAb) against the spike protein of SARS-CoV-2 was a landmark in the treatment of COVID-19 in high-risk patients [26]. In several large studies, anti-SARS-CoV-2 mAb have been shown to be highly effective in preventing the progression of COVID-19, especially when administered in the early stages of the disease [27,28]. However, despite the availability and efficacy of novel vaccines and COVID-19-targeted therapeutics, the emergence of new variants of SARS-CoV-2 continues to be a major challenge [29]. Antiviral monoclonal antibodies showed reduced efficacy against recently circulating SARS-CoV-2 variants of concern such as the Omicron B.1.1.529/BA.2 [30,31,32,33]. In consequence, in the European Union most available mAb are no longer recommended and in the United States no longer authorized to treat COVID-19. Of note, while the presence of the Omicron variant has been associated with a lower severity of COVID-19 in the general population, recent data indicate that pre-diseased and immune-compromised patients remain at high risk for morbidity and mortality from COVID-19 [34,35]. Thus, rapidly available and affordable treatment options are still urgently needed. Since the development of novel antivirals or monoclonal antibodies against emerging variants may take months to years, treatment with convalescent plasma from patients that recovered from infection with the respective variants of concern may still represent a therapeutic option especially for vulnerable high-risk patients.

Thus, we evaluated the clinical efficacy of monoclonal antibody in comparison to convalescent plasma and supportive care treatment in highly vulnerable non-vaccinated COVID-19 patients.

## 2. Materials and Methods

### 2.1. Study Population and Study Design

We conducted a monocentric retrospective observational study at our university hospital to evaluate the clinical efficacy of early monoclonal antibodies and convalescent plasma treatment in a non-vaccinated cohort at high-risk for severe COVID-19. The patients were selected in the frame of a retrospective study at different phases of the pandemic. The first antiviral treatment that was available was convalescent plasma (CP) derived from people who recovered from COVID-19. Accordingly, high-risk patients were treated with CP where indicated. Subsequently, monoclonal antibodies became available and replaced convalescent plasma therapy. The control group includes patients that were hospitalized at the early stage of the pandemic, where no antiviral treatment (such as remdesivir, convalescent plasma or monoclonal antibodies) was available or patients who refused antiviral treatment. Inclusion criteria were belonging to a high-risk cohort, WHO stage at primary presentation < 6 and giving informed consent to the study. Exclusion criteria were the following: previous immunization against COVID-19 or history of virologically confirmed COVID-19, participation in a COVID-19-treatment trial or unwilling to participate in the study. The follow-up period was 30 days. 

According to WHO COVID-19 clinical progression scale the majority of patients initially presented with mild to moderated disease, none of the patients initially presented with severe disease (Table 1) [36]. 

In total, 55 patients with PCR-confirmed COVID-19 hospitalized between March 2020 and April 2021 at the University Medicine Essen, Germany were enrolled in this study (Table 1).

COVID-19 treatment was administered according to international recommended treatment protocols, patient risk factors, treatment availability and circulating variants at time point of infection.

The first group (group 1; *n* = 26 patients) received monoclonal antibody therapy according to expanded Emergency Use Authorization (EUA) criteria of the European Medical Agency (EMA) in February 2021 [37]. The group consisted of 17 women and nine men with a median age of 58 years (range 17–78) (Table 1). 16 patients had a solid organ transplant (*n* = 3 kidney; *n* = 13 lung), six patients had a history of lung disease (*n* = 1 non-small lung cancer, *n* = 1 asthma, *n* = 1 lung fibrosis and *n* = 4 stage 4 chronic obstructive pulmonary disease (COPD)), one patient had breast cancer, one cachexia and one prostate cancer. 

The second group (group 2; *n* = 11 patients) received high-titer convalescent plasma from COVID-19 recovered individuals. This group was composed of five females and six males, and the median age was 63 years (range 44–84) (Table 1). The main indication for early convalescent plasma treatment was the risk of a fulminant COVID-19 course due to immunodeficiency (*n* = 5 with solid organ transplantation: two patients with kidney transplantation, one with liver transplantation and two with lung transplantation), a severely impaired renal function (*n* = 4), a severe lung disease (stage 4 COPD (*n* = 1)) or cancer (*n* = 1). The median neutralizing antibody titer was determined with a cell culture-based neutralization test. Only convalescent plasmas with a neutralizing antibody titer ratio of at least 1:160 were administered. The decision for convalescent plasma therapy was based solely on clinical indication and the therapy was initiated within the first 48 h after a PCR confirmed SARS-CoV-2 infection.

The third group of 18 patients (group 3) received the best supportive care at the early beginning of the pandemic when targeted antiviral therapy against SARS-CoV-2 was not available and immunomodulatory therapy was not proven for COVID-19. The group included eight women and ten men, and the median age was 75 years (range 35–93) (Table 1). Two had lung transplantation, four had kidney transplantation, four had stage 4 COPD, one had rheumatoid arthritis, one had a hematopoietic stem cell transplantation, one had esophagus cancer, one had non-small lung cancer, one had Hodgkin lymphoma, one had non-Hodgkin lymphoma, one had follicular lymphoma, and one had hepatocellular cancer. Severe COVID-19 was defined according to WHO progression scale as pneumonia with oxygen requirement. The primary outcome was all-cause in-hospital mortality within 30 days of admission, the secondary outcome was the progression of COVID-19. 

### 2.2. Therapy Regiments

**Figure 1:**
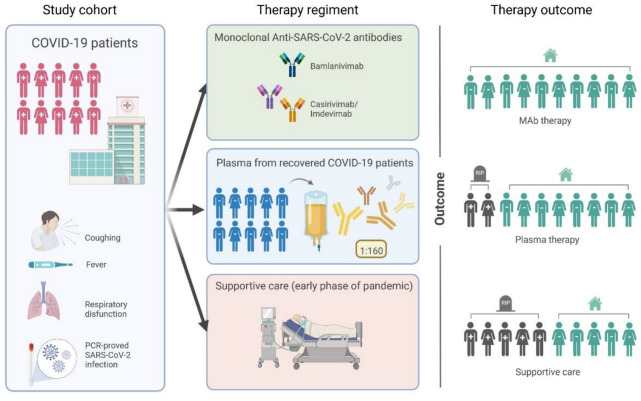
Study overview. Vulnerable patients at high risk for a severe course of COVID-19 were enrolled in the present study. The efficacy of monoclonal antibody treatment and convalescent plasma treatment was investigated and compared with best supportive care. As outcome parameter, 30-day mortality was assessed.

In the present study, we focused on the 30-day survival of COVID-19 patients that were treated with SARS-CoV-2 neutralizing monoclonal antibodies or convalescent plasma and compared it with survival rates of patients receiving supportive medical care due to the lack of specific antiviral treatment in the early phase of the pandemic (Figure 1).

#### 2.2.1. Monoclonal Antibody Therapy

Group 1, consisting of 26 patients, was treated with monoclonal anti-SARS-CoV-2 antibodies according to expanded EUA criteria of the EMA [37]. Antibody therapy was adjusted depending on the susceptibility of the circulating variants towards the respective antibodies. 23 patients received 700 mg bamlanivimab at a concentration of 35 mg/mL. Due to the increase in the locally circulating variant B.1.5 which was resistant to monotherapy with 700 mg bamlanivimab at a concentration of 35 mg/mL, three patients received 1200 mg casirivimab/imdevimab at a concentration of 120 mg/mL each. The therapy was administered according to the manufacturer’s recommendation. 

#### 2.2.2. Treatment with High-Titer Convalescent Plasma

The 11 patients from group 2 were treated with plasma from COVID-19-recovered individuals. COVID-19 convalescent plasma collection and transfusion were performed according to EU guidance [38]. The acquisition of potential plasma donors and plasmapheresis were described previously [39,40].

The plasma units that had a neutralization titer of at least 1:160 were administered to patients in an AB0-compatible manner. Neutralizing antibody titers were determined as previously described [34]. In 10 of 11 patients, therapy was performed with two plasma units of 200–280 mL each on day 1 and 3; in one patient, a third unit was applied on day 5. 

#### 2.2.3. Best-Supportive Care

The 18 patients from group 3 became SARS-CoV-2 infected early at the beginning of the pandemic. At that time, no approved therapies against SARS-CoV-2 infection existed. 

### 2.3. Ethics

The study was approved by the local ethics committee and was performed in accordance with the ethical standards noted in the 1964 Declaration of Helsinki and its later amendments or comparable ethics standards (approval no. 20-9665-BO). The production of the convalescent plasma was conducted under the permission of the county government (AZ24.05.05.02). All volunteers and patients/their legal guardians provided informed consent to participate in the study.

### 2.4. Statistics

Statistical analyses were performed using GraphPad Prism 9 (GraphPad Software, San Diego, CA, USA) software. 30-day mortality was estimated by the Kaplan–Meier method and compared with the Chi-square test. Multivariate linear models were used to determine the effects of the three different treatments and the previously described risk factors at baseline on the severity of COVID-19 outcomes. A two-sided *p*-value of 0.05 or lower was considered statistically significant.

## 3. Results

### 3.1. Study Overview

In the present study, we investigated the 30-day survival of COVID-19 patients that were treated with SARS-CoV-2 neutralizing antibodies or convalescent plasma and compared it with survival rates of patients receiving supportive medical care due to the lack of specific antiviral treatment in the early phase of the pandemic. In total, 55 patients with a PCR-confirmed SARS-CoV-2 infection were included in the study. Of them, 26 were treated with SARS-CoV-2 neutralizing antibodies bamlanivimab or casirivimab/imdevimab, 11 with convalescent plasma, and 18 received the best supportive medical care (Figure 1). Monoclonal antibodies were applied according to manufacturer instructions. Convalescent plasma treatment was conducted with two plasma units of 200–280 mL each on day 1 and 3 in 10 of 11 patients; in one patient, a third unit was given on day 5. The average neutralizing titer of convalescent plasmas used was 1:160. The survival rate was analyzed for a period of 30 days after hospitalization.

### 3.2. Antiviral Immunotherapy with Monoclonal Antibodies or Convalescent Plasma Reduced the 30-Day Mortality in High-Risk COVID-19 Patients

**Figure 2:**
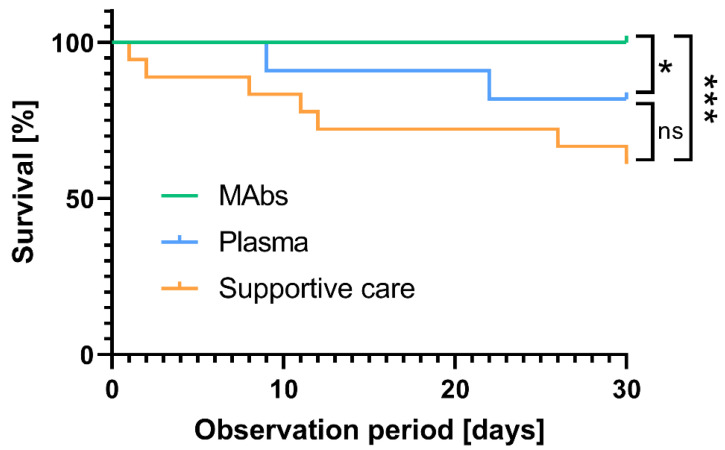
Overall survival of COVID-19 patients received either mAbs, convalescent plasma or supportive care. The survival of the patients was monitored for 30 days after initiating the treatment. Differences in the overall survival at day 30 were calculated using the Chi-square test. Comparisons were considered significant at * *p* < 0.05 and *** *p* < 0.001.

The efficacy of monoclonal antibody treatment with bamlanivimab or casirivimab/imdevimab and convalescent plasma treatment was investigated and compared with best supportive care. Patient outcome was observed for a period of 30 days upon hospitalization. All patients that were treated with monoclonal antibodies survived. Nine out of 11 patients (82%) receiving convalescent plasma treatment and eleven out of 18 (61%) of patients that received the best supportive care survived (Figure 2). Overall, the survival was significantly higher in patients who received monoclonal antibodies when compared to convalescent plasma treatment (*p* = 0.03) or best supportive care (*p* < 0.0001) (Figure 2). 

Notably, the survival was 21% higher in patients treated with convalescent plasma compared to the best supportive care group (*p* = 0.2) (Figure 2). In the mAb group severe respiratory disease developed in 1 of 26 patients (3.8%), in the convalescent plasma group in 6 of 11 (54.5%) and in the best supportive care group in 12 of 18 patients (66.7%) (*p* < 0.0001). To assess the impact on the severity of COVID-19, as defined by the most severe WHO COVID-19 score, in our cohort by the known risk factor being age, sex, Charlson Comorbidity Index (CCI), WHO COVID-19 severity on presentation and treatment group 1–3 membership, multivariable linear regression analysis was performed. In this analysis for COVID-19 severity outcome, patients’ membership in treatment group 1 (mAb, reference group) significantly influenced the COVID-19 severity score compared with the convalescent plasma group and supportive care group. In addition, only the COVID-19 severity score at presentation was associated with a more severe COVID-19 score (Table 2). The R² value for this model was 0.42.

## 4. Discussion

In the present study, we evaluated 30-day survival in differently treated unvaccinated COVID-19 patients at high risk for severe disease outcome. Depending on the availability of therapeutic options, three treatment groups could be defined: one cohort treated with monoclonal antibodies (bamlanivimab or a combination of casirivimab and imdevimab), a second cohort received convalescent plasma treatment from recovered COVID-19 individuals and a third cohort received best supportive care at the early beginning of the pandemic when no antiviral treatment against SARS-CoV-2 was available.

We demonstrated that early antiviral treatment with monoclonal antibodies is associated with a better survival rate in high-risk patients in the real-world setting compared with convalescent plasma treatment or best supportive care. Our data are in line with prior studies. Meanwhile, the therapy with mAbs in early-stage SARS-CoV-2 infections in high-risk groups is widely accepted, and clinical trials with mAbs have reported overall lower hospitalization rates in patients with mild-to-moderate COVID-19 [41,42,43]. However, previous studies have demonstrated that not all approved mAbs are suitable for treating the different variants of SARS-CoV-2 such as Omicron [44,45]. It became apparent that the development of new monoclonal antibodies is in a permanent race with the emergence of new SARS-CoV-2 variants [30,46]. 

In the present study, convalescent plasma treatment was associated with a considerably higher survival rate of 82%, compared with the best supportive care cohort (61%). Many studies have demonstrated the benefits of treatment with convalescent plasma such as reduced mortality and shorter hospitalization time [47,48]. However, it also became evident that both the timing of plasma administration as well as the neutralizing antibody titer of the plasma administered are critical for patient outcome [49,50,51]. Studies have shown that the administration of plasma in very severe courses of COVID-19 is no longer beneficial and does not reduce mortality [51,52,53].

The higher average age of patients from group 3 (supportive care) is a limitation of the study, since higher age (>60) is commonly known as a risk factor for increased mortality in COVID-19. Accordingly, older patients were the first who became vaccinated in Germany, and thus were protected from a lethal and severe course of disease. As a consequence, predominantly people around 60 were treated for COVID-19 when convalescent plasma and monoclonal antibodies became available.

Besides age, both primary and secondary immunosuppression due to inborn errors of immunity, organ transplantation, cancer treatment or other immunomodulatory treatment represents a risk factor for a severe course of COVID-19 [2]. 

Early treatment with convalescent plasma or monoclonal antibodies as well as antivirals may improve the course of disease and clinical outcome in those patients [14,54,55]. 

Both mAbs and convalescent plasma therapy show a significantly higher survival than best supportive care alone in high-risk patients. Monoclonal antibodies are not effective against every variant of SARS-CoV-2, which is a major drawback. However, timing and titer are very critical in the administration of plasma. Our data points out, however, that in the absence of approved antibodies for emerging variants, early administration of convalescent plasma may make a critical difference in the survival probability of high-risk individuals.

## 5. Conclusions

Our data highlight that early targeted passive immunization with mAbs or high-titer convalescent plasma can significantly reduce 30-day mortality in high-risk patients. Clinicians should consider early treatment with high-titer convalescent plasma as an alternative option for COVID-19-specific therapy for high-risk patients if other therapeutic options are unavailable. In addition, convalescent plasma may be a therapeutic option for infections with newly emerging SARS-CoV-2 variants that are less susceptible to approved monoclonal antibodies.

## Figures and Tables

**Table 1:** Demographic and clinical overview of the study cohort.

Group	Treated with mAB (1)	Treated with Plasma (2)	Supportive Care (3)
**Total number**	26	11	18
**Sex (w/m)**	17/9	5/6	8/10
**Median age [years]**	58 (17–78)	63 (44–84)	75 (35–93)
**Mean Charlson Comorbidity Index Score, ±SD**	3.23 ± 2.0	6.54 ± 2.7	5.0 ± 2.12
**Severely immunocompromised Patients, *n* (%)**	17 (65%)	7 (63%)	12 (66%)
**History of pulmonary disease, *n* (%)**	19 (73%)	3 (27%)	5 (27%)
**Predominant circulating variant?**	Alpha B.1.1.7	Alpha B.1.1.7	D614G and Alpha B.1.1.7
**Range of admission to hospital**	02.2021 to 04.2021	07.2020 to 11.2020	03.2020 to 02.2021
**Number of Patient with moderate disease**	5 (19%)	3 (27%)	3 (16%)
**Progression to severe COVID-19 defined by WHO score ≥ 6, *n* (%)**	1 (4%)	6 (54%)	12 (66%)

**Table 2:** Results of the regression model indicating the determinants of the COVID-19 severity defined by WHO score. Comparisons were considered significant at * *p* < 0.05 and ** *p* < 0.01. CCI = Charlson index; G1–3 = group 1–3; mAb = monoclonal antibody therapy; SC = supportive care.

Parameter	Standardized Coefficients ß	95% CI	*p* Value
Sex	0.371	−0.8074 to 1.550	0.53
Age	−0.0027	−0.07792 to 0.02377	0.29
CCI	0.139	−0.1187 to 0.3968	0.28
WHO COVID-19 severity score at presentation	0.81	0.01247 to 1.605	0.04 *
G1 mAb vs. G2 Plasma	2.002	0.3113 to 3.693	0.021 *
G1 mAb vs. G3 SC	3.318	1.754 to 4.881	<0.0001 **

## Data Availability

The data presented in this study are available on request from the corresponding author.
